# Adoption of Health Information Technologies by Area Socioeconomic Deprivation Among US Hospitals

**DOI:** 10.1001/jamahealthforum.2025.3035

**Published:** 2025-09-05

**Authors:** Alice S. Yan, Nate C. Apathy, Jie Chen

**Affiliations:** 1 Department of Health Policy and Management, School of Public Health, University of Maryland, College Park; 2 Hospital and Public Health Interdisciplinary Research (HAPPY) Lab, School of Public Health, University of Maryland, College Park; 3 University of Maryland Center on Aging, School of Public Health, University of Maryland, College Park; 4 Regenstrief Institute, Indianapolis, Indiana; 5 University of Maryland Institute for Health Computing, North Bethesda

## Abstract

**Importance:**

Access to and quality of care vary substantially by area socioeconomic status. Expanding hospital health information technology (HIT) adoption may help reduce these disparities, given hospitals’ central role in serving underserved populations.

**Objective:**

To examine variations in US hospital adoption of telehealth and health information exchange (HIE) functionalities by hospital service area (HSA) socioeconomic deprivation.

**Design, Setting, and Participants:**

This cross-sectional study links data from the 2018-2023 American Hospital Association Annual Survey and Information Technology Survey with HSA-level area deprivation index. Nonfederal acute care hospitals with complete data on HIT outcomes, comprising 16 646 observations for the telehealth outcomes and 9218 observations for the HIE outcomes across 6 years, were included. Data were analyzed from February 2024 to February 2025.

**Exposures:**

HSA-level area deprivation index in quartiles.

**Main Outcomes and Measures:**

Hospital adoption of treatment-stage telehealth and postdischarge telehealth services and HIE infrastructure supporting electronic data query and availability. Descriptive, regression, and Blinder-Oaxaca decomposition analyses and visualized time trends in hospital HIT adoption were used in analyses.

**Results:**

This study included 16 646 hospital-level observations and 9218 observations for health information exchange functionalities. Hospitals in the most socioeconomically deprived HSAs were significantly less likely to adopt HIT compared with those in the least deprived areas (treatment-stage telehealth: marginal effect [ME], −0.03; 95% CI, −0.06 to −0.01; postdischarge telehealth: ME, −0.03; 95% CI, −0.07 to 0.01; electronic data query capability: ME, −0.03; 95% CI, −0.06 to −0.01; electronic data availability: ME, −0.06; 95% CI, −0.11 to −0.01). Year fixed effects indicated significant increases in HIT adoption from 2018 to 2023, regardless of HSA deprivation level. Decomposition analyses showed that differences in hospital bed size, urban/rural location, and accountable care organization participation explained a substantial portion of the disparities by HSA deprivation.

**Conclusions and Relevance:**

In this study, hospitals in more socioeconomically disadvantaged HSAs remained likely to adopt telehealth and HIE functionalities. Nevertheless, HIT adoption has grown steadily over time. Accountable care organization participation may support HIT infrastructure and help reduce geographic disparities in adoption and access to care.

## Key Points

**Question:**

How does hospital adoption of health information technology (HIT) in the US vary by area socioeconomic deprivation?

**Findings:**

In this cross-sectional study including 16 646 hospital-level observations and 9218 observations for health information exchange functionalities, using 2018-2023 pooled data of nonfederal acute care hospitals, those in the most deprived areas were significantly less likely to adopt HIT, including treatment-stage telehealth, postdischarge telehealth, electronic data query capability, and data availability. Adoption increased over time across all hospitals and deprivation levels.

**Meaning:**

Hospitals in under-resourced communities may not fully realize the benefits of HIT-enabled care, but targeted policies may help close geographic adoption gaps.

## Teaser

This cross-sectional study examines variations in US hospital adoption of telehealth and health information exchange functionalities by hospital service area socioeconomic deprivation.

## Introduction

Hospital-based health information technology (HIT) functionalities—such as telehealth-assisted treatment and postdischarge measures, health information exchanges (HIEs), and electronic data exchanges—have been shown to improve timely access to care, especially in remote areas without nearby health centers.^1,2,3^ Efficient HIE systems can also reduce redundancy in medical procedures and mitigate care fragmentation, particularly for hospitals serving rural patients and patients insured by Medicare and Medicaid.^2^ In addition, hospital-based HIT has the potential to reduce health disparities by benefiting underserved communities.^1,2,4^

However, persistent challenges, such as limited technological infrastructure, insufficient funding, and lack of training opportunities, continue to hinder HIT adoption in under-resourced communities.^5,6^ Telehealth services, such as telehealth-assisted postcare management, are especially difficult to implement in areas with lower socioeconomic status, where infrastructure gaps and scarce community resources present major barriers.^7^ These limitations contribute to ongoing disparities in care access, quality, and outcomes.^8,9,10^ Similarly, hospitals serving disadvantaged communities are less likely to adopt HIE systems.^2,9^ Socioeconomic deprivation consistently predicts lower hospital engagement in interoperable data exchange, especially where community partners lack the capacity to share or act on electronic health information.^2,5,6^ Moreover, HIT functionalities, such as virtual visits and chronic care management, depend on patients’ access to digital tools.^5,6,8^ In high-deprivation areas, limited broadband, low digital literacy, and language barriers continue to restrict equitable HIT use.^2,8^

In response to these challenges, a range of policies have been introduced to expand telehealth services and strengthen HIE infrastructure in underserved areas. The Coronavirus Aid, Relief, and Economic Security (CARES) Act provided funding for telehealth expansion across rural hospitals in response to the COVID-19 pandemic, while the Trusted Exchange Framework and Common Agreement (TEFCA) established a framework for nationwide interoperable HIE between health care organizations.^2,11^ To further address the financial and workforce barriers faced by socioeconomically disadvantaged hospitals, alternative payment models and workforce development initiatives may offer more sustainable solutions.^9^

Given persistent challenges and encouraging recent policy efforts to promote digital health equity, this is a timely moment to generate updated evidence on hospital HIT adoption. Our study examined recent trends using a measure of geographic variation that more comprehensively reflects community-level social determinants of health. As noted above, socioeconomically deprived neighborhoods and the health care organizations that serve them often lack the technological infrastructure for telehealth and HIE due to high development and operating costs.^5,6^ When area-level socioeconomic deprivation is correlated with lower HIT adoption, under-resourced hospitals may underinvest in these tools, limiting their potential to improve patient care.^2,9^ Hence, we hypothesized that hospitals located in more socioeconomically deprived areas are less likely to adopt telehealth and HIE functionalities compared with those in more advantaged areas.

## Methods

### Data Sources

Our study leveraged the 2018-2023 American Hospital Association (AHA) Annual Survey and associated Information Technology (IT) Survey.^12,13^ We linked the AHA data with the 2021 area deprivation index (ADI) for each hospital service area (HSA).^14^ We further linked these data with the 2023 Area Health Resources File to access additional covariates.^15^ The ADI is a measure constructed from 17 US census variables encompassing educational attainment, employment, housing quality, and poverty.^16^ It has been widely used to assess various neighborhood-level health outcomes, as well as validated by recent studies.^17,18^ This study followed the Strengthening the Reporting of Observational Studies in Epidemiology (STROBE) reporting guideline and was exempt from institutional review board approval with a waiver of informed consent, owing to the use of deidentified data per 45 CFR §46.

Our study focused on nonfederal acute care hospitals, which are commonly distinguished from federal and specialty/long-term care hospitals in both the Office of the National Coordinator for Health Information Technology definitions and the broader literature.^2,19^ These hospitals deliver most of the health care in US and operate under different incentives for HIT adoption. In contrast, federal and specialty/long-term care hospitals serve distinct populations and are subject to unique ownership structures and policy levers.^2,20^ Given these differences, nonfederal acute care hospitals were the appropriate focus for this analysis.

### Measurement

#### Outcomes

Our study examined 4 hospital-based HIT functionalities. The first was a treatment-stage telehealth indicator, equaling 1 if a hospital used telemedicine for consultation and office visits, the intensive care unit, stroke care, and psychiatric/addiction treatment or equaling 0 otherwise. The second was a postdischarge telehealth indicator, equaling 1 if a hospital used remote patient monitoring for both postdischarge and ongoing chronic care management or equaling 0 otherwise. These 2 measures were obtained from the 2018-2023 AHA Annual Survey. We also used 2 dichotomous measures of HIE adoption. The first, electronic data query capability, was coded as 1 if a hospital could electronically query patient health data from external health care professionals and coded as 0 otherwise. The second, electronic data availability, was coded as 1 if clinical data from external health care professionals was electronically available to the hospital and coded as 0 otherwise. These measures were derived from the 2018-2020 and 2022-2023 AHA IT Surveys; the survey was not administered in 2021. We used dichotomous HIT measures to align with Office of the National Coordinator for Health Information Technology standards and prior literature, allowing for consistent comparisons across hospitals.^19,21^ eMethods 1 in Supplement 1 provides more details on how AHA Annual and IT Survey variables were mapped to these measures.

#### Exposures

We used a Dartmouth Atlas zip code to HSA crosswalk and population data from Geocorr to calculate a population-weighted average of zip code–level ADI percentiles, producing an HSA-level ADI measure.^14,22,23^ We used HSA as the geographic unit to assess variation in hospital HIT adoption, as HSAs represent self-contained regions—typically a county or cluster of contiguous counties—served by a particular hospital or hospital group and are more granular than a hospital referral region.^24,25^ We grouped the HSA-level ADI percentiles into quartiles, from least (first quartile) to most (fourth quartile) socioeconomically disadvantaged.

#### Covariates

We also controlled for other hospital characteristics commonly associated with HIT adoption, including control type (not-for-profit, for-profit, and government-owned hospitals), bed size, urban/rural geography, teaching designation, and percentage of county population by race and ethnicity (sourced from the Area Health Resources File).^21,26^ Given evidence that hospitals participating in accountable care organizations (ACOs) linked to higher HIT adoption, we also included ACO participation as a covariate.^27,28^

### Statistical Analysis

We first compared descriptive statistics for 4 HIT functionalities by HSA deprivation index national quartile for 2023 and visualized the results using a bar chart. We also presented temporal trends in unadjusted hospital adoption rates for each functionality from 2018 to 2023. To estimate associations between HSA-level socioeconomic deprivation and HIT adoption, we conducted logistic regression analyses. We reported marginal effects (MEs) with 95% CIs using robust SEs clustered at the hospital level to account for within-hospital autocorrelation over time. We also applied nonresponse weights for the HIE outcomes based on the probability that a hospital in the AHA Annual Survey responded to the AHA IT Survey, conditional on hospital characteristics. These weights adjust for observed differences between respondents and the broader hospital population. Finally, to quantify factors driving differences in HIT adoption across HSA deprivation levels, we conducted Blinder-Oaxaca decomposition analyses.^29,30,31^ These models estimated the extent to which disparities in adoption between the most socioeconomically deprived areas (fourth ADI quartile) and less deprived areas (first to third ADI quartiles) were explained by hospital characteristics included in our empirical model (eg, hospital bed size, urban/rural classification, and ACO participation).

We conducted several sensitivity analyses to test the robustness of our regression results to alternative definitions of outcomes and exposures. First, we redefined the telehealth outcomes (eTable 1 in Supplement 1), coding the treatment-stage telehealth indicator as 1 if a hospital used telemedicine for any of consultation/office visits, the intensive care unit, stroke care, or psychiatric/addiction treatment and coding the postdischarge telehealth as 1 if a hospital used remote patient monitoring for either postdischarge or ongoing chronic care management. Second, we repeated our regression analyses using hospital referral regions instead of HSAs to measure area-level deprivation (eTable 2 in Supplement 1).^14,22,23^ As expected, we observed greater variations in hospital telehealth and HIE adoption by hospital referral region due to their broader geographic scope. Third, we estimated unweighted versions of our primary HIE regressions (eTable 3 in Supplement 1). Finally, to complement the bar charts, we created maps illustrating spatial patterns in HIT adoption by HSA deprivation for the year 2023 (eFigure in Supplement 1).

All statistical analyses were performed using Stata MP, version 18 (StataCorp). Wald tests were used to calculate *P* values for all logistic regressions, while *t* tests were used for the Blinder-Oaxaca decomposition. Significance was set at *P* < .05, and all *P* values were 2-tailed. Data were analyzed from February 2024 to February 2025.

## Results

**Figure 1.**
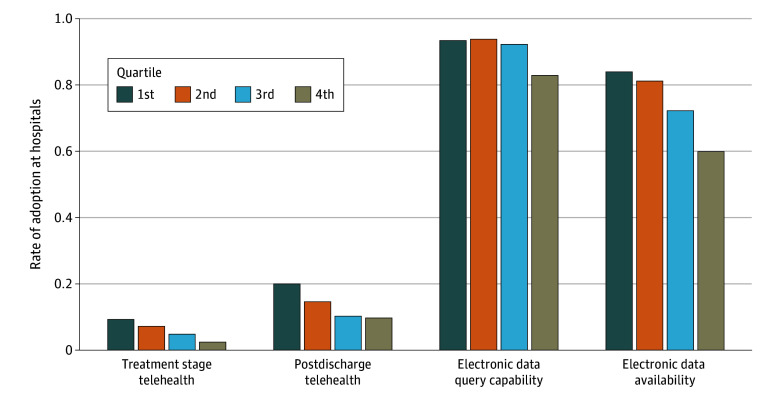
Bar Chart of Hospital Health Information Technology Adoption by Functionality and Hospital Service Area Deprivation for 2023 The study population consists of nonfederal acute care hospitals. The hospital service area deprivation index was derived from the 2021 area deprivation index. Data on hospital telehealth and health information exchange functionality adoption were sourced from the 2023 American Hospital Association Annual Survey and 2023 American Hospital Association Annual IT Survey, respectively.

The final sample included 16 646 hospital-level observations for treatment-stage telehealth and postdischarge telehealth and 9218 observations for HIE functionalities. eMethods 2 in Supplement 1 provides a breakdown of observations by survey year. Descriptive statistics (eTable 4 in Supplement 1) showed an inverse association between HSA-level socioeconomic deprivation and hospital adoption of the HIT functionalities studied (Figure 1). Hospitals in the least-deprived areas (first ADI quartile) had significantly higher adoption rates across all HIT measures compared with those in the most deprived areas (fourth ADI quartile). Additional summary statistics are provided in eTable 4 in Supplement 1.

**Figure 2.**
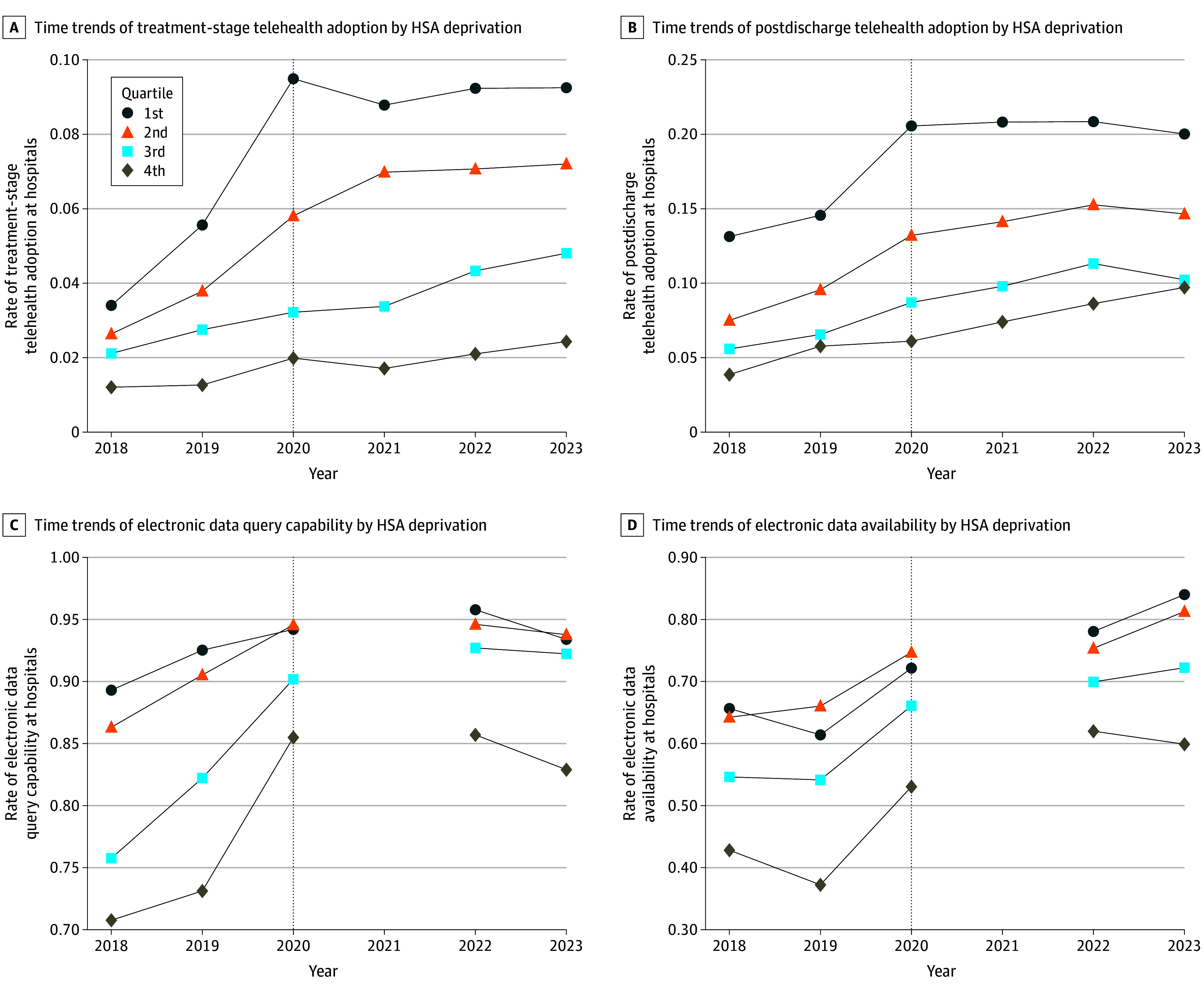
Time Trends of Hospital Telehealth and Health Information Exchange Adoption by Hospital Service Area (HSA) Deprivation The study population consists of nonfederal acute care hospitals. The HSA deprivation index was derived from the 2021 area deprivation index. Data on hospital telehealth functionality adoption were sourced from the 2018-2023 American Hospital Association Annual Survey. Data on hospital health information exchange functionality adoption were obtained from the 2018 to 2020 and 2022 to 2023 American Hospital Association Annual Information Technology Survey. The 2021 American Health Association Annual Information Technology Survey was not implemented.

Time trends (Figure 2) showed consistently lower unadjusted HIT adoption rates among hospitals in more socioeconomically deprived HSAs across all functionalities and years. Adoption was highest for HIE infrastructure (electronic data query capability and availability) compared with treatment-stage telehealth and postdischarge telehealth. From 2018 to 2023, adoption increased across all HIT measures and deprivation levels, although telehealth adoption appeared to plateau after the COVID-19 pandemic.

Regression analyses (Table 1) showed that hospitals in the most socioeconomically deprived HSAs were significantly less likely to adopt all 4 HIT functionalities compared with those in the least deprived areas (treatment-stage telehealth: ME, −0.03; 95% CI, −0.06 to −0.01; postdischarge telehealth: ME, −0.03; 95% CI, −0.07 to 0.01; electronic data query capability: ME, −0.03; 95% CI, −0.06 to −0.01; electronic data availability: ME, −0.06; 95% CI, −0.11 to −0.01). ACO participation emerged as a strong predictor of HIT adoption, with higher adoption probabilities across all outcomes (treatment-stage telehealth: ME, 0.02; 95% CI, 0.01 to 0.03; postdischarge telehealth: ME, 0.03; 95% CI, 0.01 to 0.05; electronic data query capability: ME, 0.05; 95% CI, 0.04 to 0.07; electronic data availability: ME, 0.07; 95% CI, 0.04-0.10). Survey year indicators showed increasing HIT adoption over time. Compared with 2018, adoption in 2023 was significantly higher across all functionalities (treatment-stage telehealth: ME, 0.04; 95% CI, 0.03 to 0.05; postdischarge telehealth: ME, 0.06; 95% CI, 0.05 to 0.07; electronic data query capability: ME, 0.07; 95% CI, 0.05 to 0.09; electronic data availability: ME, 0.17; 95% CI, 0.15 to 0.20).

**Table 1.** Marginal Effects (MEs) of Hospital Service Area (HSA) Deprivation on Hospital Telehealth and Health Information Exchange (HIE) Adoption^a^

Characteristic	ME (95% CI)			
	Telehealth adoption (full vs partial/none)		HIE adoption (weighted)	
	Treatment stage (n = 16 646)^b^	Postdischarge (n = 16 646)^b^	Electronic query capability (n = 9218)^b^	Electronic availability (n = 9218)^b^
HSA deprivation index national quartile				
Quartile 1 (least deprived)	0 [Reference]	0 [Reference]	0 [Reference]	0 [Reference]
Quartile 2	−0.01 (−0.03 to 0.01)	−0.04 (−0.06 to −0.01)^c^	0.01 (−0.01 to 0.03)	0.02 (−0.02 to 0.05)
Quartile 3	−0.02 (−0.04 to 0)^d^	−0.04 (−0.07 to −0.01)^d^	−0.02 (−0.05 to 0)	−0.02 (−0.06 to 0.02)
Quartile 4 (most deprived)	−0.03 (−0.06 to −0.01)^c^	−0.03 (−0.07 to 0.01)	−0.03 (−0.06 to −0.01)^d^	−0.06 (−0.11 to −0.01)^d^
Hospital ACO participation				
Not in an ACO	0 [Reference]	0 [Reference]	0 [Reference]	0 [Reference]
In a Medicare, Medicaid, or private ACO	0.02 (0.01 to 0.03)^c^	0.03 (0.01 to 0.05)^e^	0.05 (0.04 to 0.07)^e^	0.07 (0.04 to 0.10)^e^
Hospital control type				
Not for profit	0.01 (−0.01 to 0.03)	0.02 (−0.01 to 0.04)	0.06 (0.04 to 0.09)^e^	0.20 (0.16 to 0.24)^e^
For profit	−0.03 (−0.05 to 0)^d^	−0.10 (−0.13 to −0.06)^e^	−0.05 (−0.09 to −0.01)^d^	0.14 (0.07 to 0.20)^e^
Government	0 [Reference]	0 [Reference]	0 [Reference]	0 [Reference]
Hospital bed size				
Small (<50 beds)	−0.02 (−0.04 to −0.01)^c^	−0.04 (−0.06 to −0.02)^e^	−0.04 (−0.06 to −0.01)^c^	0.01 (−0.03 to 0.04)
Medium (50-199 bed)	0 [Reference]	0 [Reference]	0 [Reference]	0 [Reference]
Large (≥200 beds)	0.01 (0 to 0.03)	0.09 (0.06 to 0.13)^e^	0.04 (0.01 to 0.06)^c^	0.03 (−0.01 to 0.07)
Hospital geography				
Metropolitan	0.02 (0 to 0.04)	0.02 (−0.01 to 0.06)	0.04 (0.02 to 0.07)^c^	0.13 (0.08 to 0.18)^e^
Micropolitan	0.05 (0.02 to 0.07)^e^	0.05 (0.01 to 0.09)^d^	0.04 (0.02 to 0.07)^c^	0.04 (−0.01 to 0.09)
Rural	0 [Reference]	0 [Reference]	0 [Reference]	0 [Reference]
Hospital teaching designation				
Major teaching hospital	0.09 (0.05 to 0.13)^e^	0.21 (0.15 to 0.27)^e^	0.06 (0.02 to 0.09)^c^	0.13 (0.07 to 0.19)^e^
Minor teaching hospital	0 (−0.01 to 0.02)	0.01 (−0.01 to 0.04)	0.02 (0 to 0.04)^d^	0.03 (0 to 0.06)
Not a teaching hospital	0 [Reference]	0 [Reference]	0 [Reference]	0 [Reference]
Percentage of county population that identifies as a racial or ethnic minority group^f^	−0.02 (−0.06 to 0.01)	−0.12 (−0.18 to −0.07)^e^	−0.09 (−0.13 to −0.04)^e^	−0.18 (−0.25 to −0.10)^e^
Survey year fixed effects				
2018	0 [Reference]	0 [Reference]	0 [Reference]	0 [Reference]
2019	0.01 (0.01 to 0.02)^e^	0.02 (0.01 to 0.02)^e^	0.02 (0.01 to 0.04)^d^	−0.02 (−0.04 to 0)
2020	0.03 (0.03 to 0.04)^e^	0.05 (0.04 to 0.06)^e^	0.07 (0.05 to 0.09)^e^	0.08 (0.05 to 0.10)^e^
2021	0.04 (0.03 to 0.04)^e^	0.06 (0.05 to 0.07)^e^	NA^g^	NA^g^
2022	0.04 (0.03 to 0.05)^e^	0.07 (0.05 to 0.08)^e^	0.09 (0.07 to 0.11)^e^	0.12 (0.10 to 0.15)^e^
2023	0.04 (0.03 to 0.05)^e^	0.06 (0.05 to 0.07)^e^	0.07 (0.05 to 0.09)^e^	0.17 (0.15 to 0.20)^e^

Abbreviations: ACO, accountable care organization; NA, not applicable.

^a^ The study population consists of nonfederal acute care hospitals. MEs were estimated from logistic regressions of hospital telehealth and HIE functionality adoption on HSA deprivation, controlling for other hospital characteristics as well as survey year fixed effects. 95% CIs were based on robust SEs clustered at the hospital level, and nonresponse weights were applied. The HSA deprivation index was derived from the 2021 area deprivation index. Data on telehealth functionality adoption and other hospital characteristics were sourced from the 2018 to 2023 American Health Association Annual Survey. Data on HIE functionality adoption were obtained from the 2018 to 2020 and 2022 to 2023 American Health Association Annual Information Technology Survey. Data on county population by race and ethnicity were obtained from the 2023 Area Health Resources File.

^b^ Sample sizes are cumulative over the study period (2018 to 2023), and differences are due to varying response rates to the underlying survey questions regarding telehealth services and HIE infrastructure.

^c^ *P* < .01.

^d^ *P* < .05.

^e^ *P* < .001.

^f^ Racial or ethnic minority group includes Hispanic ethnicity and non-Hispanic American Indian or Alaska Native, non-Hispanic Asian, non-Hispanic Black, non-Hispanic multiracial, non-Hispanic Native Hawaiian or Other Pacific Islander, and non-Hispanic other race and excludes non-Hispanic White race.

^g^ The 2021 American Health Association Annual Information Technology Survey was not implemented.

Decomposition analyses (Table 2) showed that our model explained a substantial share of the difference in hospital HIT adoption across HSA deprivation levels: 71.7% for treatment-stage telehealth, 103.5% for postdischarge telehealth, 63.5% for electronic data query capability, and 59.8% for electronic data availability. Among hospital characteristics, bed size accounted for the largest share of the explained differences in treatment-stage telehealth adoption and postdischarge telehealth adoption. For example, large bed sizes explained 59.9% of the difference in postdischarge adoption by HSA deprivation. This was followed by urban/rural location and teaching status. ACO participation explained approximately 7.6% to 8.6% of the observed differences. Similarly, for HIE adoption, hospital bed size, urban/rural classification, and control type explained much of the observed variation. ACO participation accounted for approximately 6.9% to 8.1% of the difference in HIE adoption.

**Table 2.** Decomposition of Hospital Telehealth and Health Information Exchange (HIE) Adoption by Hospital Service Area (HSA) Deprivation^a^

Variable	Coefficient (95% CI)			
	Telehealth adoption (full vs partial/none)		HIE adoption (weighted)	
	Treatment stage (n = 16 646)^b^	Postdischarge (n = 16 646)^b^	Electronic query capability (n = 9218)^b^	Electronic availability (n = 9218)^b^
HSA deprivation index national quartile				
Quartile 1 to quartile 3	0.06 (0.05-0.07)^c^	0.14 (0.13-0.16)^c^	0.91 (0.90-0.92)^c^	0.70 (0.68-0.71)^c^
Quartile 4	0.02 (0.01-0.03)^c^	0.07 (0.05-0.09)^c^	0.79 (0.76-0.82)^c^	0.50 (0.46-0.54)^c^
Difference in coefficients	0.04 (0.03-0.05)^c^	0.07 (0.05-0.09)^c^	0.11 (0.08-0.15)^c^	0.19 (0.15-0.23)^c^
Decomposition of difference, %				
Difference explained by the model overall	71.70^c^	103.52^c^	63.53^c^	59.75^c^
Difference explained by individual factors^d^				
Hospital ACO participation (in a Medicare, Medicaid, or private ACO)	7.55^e^	8.58^c^	8.09^c^	6.93^c^
Hospital control type (not for profit)	NA^d^	NA^d^	12.18^c^	19.40^c^
Hospital bed size				
Small (<50 beds)	12.97^f^	14.63^e^	13.58^c^	NA^d^
Large (≥200 beds)	24.53^c^	59.92^c^	9.23^e^	10.58^c^
Hospital geography (urban)	16.27^f^	14.21^f^	20.54^c^	18.87^c^
Hospital teaching designation (teaching hospital)	9.67	16.03^e^	7.83^f^	8.86^e^
Percentage of county population that identifies as a racial or ethnic minority group^g^	NA^d^	−10.13^e^	−8.44^c^	−7.35^c^

Abbreviations: ACO, accountable care organization; NA, not applicable.

^a^ The study population consists of nonfederal acute care hospitals. Coefficients were estimated from Blinder-Oaxaca decompositions of hospital telehealth and HIE functionality adoption on HSA deprivation, controlling for other hospital characteristics as well as survey year fixed effects. 95% CIs were based on robust SEs clustered at the hospital level, and nonresponse weights were applied. The HSA deprivation index was derived from the 2021 area deprivation index. Data on telehealth functionality adoption and other hospital characteristics were sourced from the 2018 to 2023 American Health Association Annual Survey. Data on HIE functionality adoption were obtained from the 2018 to 2020 and 2022 to 2023 American Health Association Annual Information Technology Survey. Data on county population by race and ethnicity were obtained from the 2023 Area Health Resources File.

^b^ Sample sizes are cumulative over the study period (2018 to 2023), and differences are due to varying response rates to the underlying survey questions regarding telehealth services and HIE infrastructure.

^c^ *P* < .001.

^d^ Only those factors that contributed 5% or more to the observed difference are presented.

^e^ *P* < .01.

^f^ *P* < .05.

^g^ Racial or ethnic minority group includes Hispanic ethnicity and non-Hispanic American Indian or Alaska Native, non-Hispanic Asian, non-Hispanic Black, non-Hispanic multiracial, non-Hispanic Native Hawaiian or Other Pacific Islander, and non-Hispanic other race and excludes non-Hispanic White race.

## Discussion

Our findings showed that hospitals in the most socioeconomically deprived HSAs have lower rates and likelihoods of HIT adoption compared with those in less deprived areas. Encouragingly, regression results indicate that HIT adoption has increased over time across all hospitals, regardless of deprivation level, with notable growth since 2018. Visualizations further highlight that adoption rates for HIE infrastructure, supporting electronic data query and availability, exceed those for treatment-stage telehealth and postdischarge telehealth, even among the most under-resourced hospitals.

Hospitals in disadvantaged areas may be underinvesting in HIT due to persistent barriers, limiting their ability to realize its full benefits.^2,9^ Telehealth facilitates timely access to care in remote settings, while HIE reduces costs and care fragmentation.^1,2^ Together, these tools support population health management and can improve care equity in underserved communities.^1,2,4^ Recent evidence suggests that HIT adoption not only reduces racial, ethnic, and rural health disparities but may also help address inequities tied to regional socioeconomic status.^8,9,10^

However, numerous challenges remain. The decomposition results showed that hospital bed size and urban/rural location contributed significantly to differences in HIT adoption, consistent with prior research indicating that under-resourced hospitals are less likely to have the capacity to invest in such technologies. We speculated that limited HIT adoption and this digital divide may further undermine hospitals’ ability to serve their communities, particularly in rural or underserved areas, potentially accelerating challenges like those seen in recent rural hospital closures.^32^ While hospital closures are driven by multiple factors, this trend raises concerns about timely access to care.^32^ At the same time, our results showed that overall hospital HIT adoption has increased nationally. More research is needed to understand whether hospitals that remain in rural or underserved areas are investing in HIT and how this may impact health equity and care delivery.

This study highlights the potential of value-based payment models, particularly ACOs, for promoting adoption of telehealth and HIE functionalities.^27,28,33^ Our results suggest that ACO participation may enhance HIT infrastructure and is associated with higher telehealth and HIE adoption, even in socioeconomically disadvantaged HSAs. ACO-participating hospitals are incentivized to reduce costs and improve care delivery, often resulting in broader use of HIT services and greater investment in technology.^27,34,35^ One survey found that 31% of ACO-affiliated hospital emergency departments adopted telehealth and 65% invested in HIE to support care coordination.^27^ ACO participation has also been linked to reduced Medicare spending and improved care.^36^ Despite these benefits, ACO adoption remains limited in underserved areas.^37,38^

Beyond our empirical findings, several persistent structural and operational barriers continue to hinder HIT adoption in under-resourced settings. Workforce shortages, insufficient reimbursement for telehealth services, limited EHR interoperability, and vendor-imposed restrictions on HIE use all present significant challenges.^5,6,7^ These obstacles reinforce the digital divide and disproportionately affect marginalized populations, including racial and ethnic minority individuals and older adults.^5,39^ To close these gaps, policy interventions must go beyond incentives for adoption. Investments in workforce development, reforms to reimbursement structures, and strengthened technical standards for interoperability are essential.^9^ Without targeted strategies to address these underlying barriers, hospitals in disadvantaged areas may remain unable to fully leverage HIT for care coordination, population health management, and equitable delivery of services.

### Limitations

Our study has several limitations. First, its cross-sectional design allows for the identification of associations but not causal inferences. Second, reliance on self-reported data from the AHA Annual and IT Surveys introduces potential recall and nonresponse bias due to low response rates.^40,41^ Third, we used the 2021 ADI to measure socioeconomic deprivation, limiting our ability to assess changes over time, although such variation is likely minimal. The absence of the 2021 IT Survey also leaves a gap in the trend analysis.^14^ Fourth, our results showed that hospital ACO participation was associated with a higher rate of HIT adoption. Future research could build on this finding by examining specific ACO characteristics, such as financial risk level or organization type, to better understand how ACO design can support HIT adoption and enhance patient care across diverse settings. Furthermore, while we focused on nonfederal acute care hospitals, HIT disparities in federal, specialty, and long-term care hospitals have grown increasingly relevant and warrant further research. Finally, our study examined HIT adoption but did not assess HIT utilization or the influence of state policies, both of which are critical areas for future investigation.

## Conclusions

In this cross-sectional study, hospitals in more socioeconomically disadvantaged HSAs were less likely to adopt telehealth and HIE functionalities. However, adoption has increased over time, offering reason for optimism. Importantly, ACO participation appears to support telehealth and HIE infrastructure development, even in under-resourced settings. To promote equitable access to digital health tools, policy efforts should focus on addressing socioeconomic barriers and expanding ACO participation in disadvantaged communities, creating the conditions necessary for broader, more equitable HIT adoption.

## Notes

Supplement 1. **eMethods 1.** Mapping of AHA Survey Questions to Telehealth and HIE Adoption Outcomes **eMethods 2.** Sample Sizes for Telehealth and HIE Adoption Outcomes by Survey Year **eTable 1.** Marginal Effects of HSA Deprivation on Hospital Telehealth Adoption **eTable 2.** Marginal Effects of HRR Deprivation on Hospital Telehealth and HIE Adoption **eTable 3.** Marginal Effects of HSA Deprivation on Hospital HIE Adoption **eTable 4.** Hospital and County Characteristics Overall and by Subgroup for 2023 **eFigure.** Maps of Hospital Telehealth and HIE Adoption and HSA Deprivation for 2023

Supplement 2. Data Sharing Statement
