# Supplementary material for: Adoption of Health Information Technologies by Area Socioeconomic Deprivation Among US Hospitals
Source: JAMA Health Forum. 2025 Sep 5;6(9):e253035. doi: 10.1001/jamahealthforum.2025.3035 (PMC12413650; doi:10.1001/jamahealthforum.2025.3035)
Supplement: Supplement 1. — eMethods 1. Mapping of AHA Survey Questions to Telehealth and HIE Adoption Outcomes eMethods 2. Sample Sizes for Telehealth and HIE Adoption Outcomes by Survey Year eTable 1. Marginal Effects of HSA Deprivation on Hospital Telehealth Adoption eTable 2. Marginal Effects of HRR Deprivation on Hospital Telehealth and HIE Adoption eTable 3. Marginal Effects of HSA Deprivation on Hospital HIE Adoption eTable 4. Hospital and County Characteristics Overall and by Subgroup for 2023 eFigure. Maps of Hospital Telehealth and HIE Adoption and HSA Deprivation for 2023 [file jamahealthforum-e253035-s001.pdf]

Supplemental Online Content

Yan AS, Apathy NC, Chen J. Adoption of health information technologies by area socioeconomic deprivation among US hospitals. *JAMA Health Forum*. 2025;6(9):e253035. doi:10.1001/jamahealthforum.2025.3035

eMethods 1. Mapping of AHA Survey Questions to Telehealth and HIE Adoption Outcomes

eMethods 2. Sample Sizes for Telehealth and HIE Adoption Outcomes by Survey Year

eTable 1. Marginal Effects of HSA Deprivation on Hospital Telehealth Adoption

eTable 2. Marginal Effects of HRR Deprivation on Hospital Telehealth and HIE Adoption

eTable 3. Marginal Effects of HSA Deprivation on Hospital HIE Adoption

eTable 4. Hospital and County Characteristics Overall and by Subgroup for 2023

eFigure. Maps of Hospital Telehealth and HIE Adoption and HSA Deprivation for 2023

This supplementary material has been provided by the authors to give readers additional information about their work.

© 2025 Yan AS et al. *JAMA Health Forum*.

eMethods 1. Mapping of AHA Survey Questions to Telehealth and HIE Adoption Outcomes

Telehealth Adoption Outcomes

Indicator for treatment-stage telehealth:

= 1 (“Yes”) if response 2A was checked for items 1A-1D in the following survey question
= 0 (“No”) otherwise

Indicator for post-discharge telehealth:

= 1 (“Yes”) if response 2A was checked for both items listed under 1E in the following survey question
= 0 (“No”) otherwise

AHA Annual Survey question on telehealth services:

For each service listed below (1), please check all the categories that describe how the service is provided (2).

1. List of telehealth services:
    A. Consultations and office visits
    B. Electronic intensive care unit (eICU)
    C. Stroke care
    D. Psychiatric and addiction treatment
    E. Remote patient monitoring
        I. Post-discharge
        II. Ongoing chronic care management

2. Categories of service delivery (1 checkbox per category, for each of the services listed above):
    A. Provided through the hospital or a subsidiary
    B. Provided through the health system
    C. Provided through a joint venture or other contractual arrangement
    D. Not provided

HIE Adoption Outcomes

Indicator for electronic data query capability:

= 1 (“Yes”) if response 1 or 2 was checked for the following survey question
= 0 (“No”) otherwise

AHA IT Survey question on electronic query of data:

Does your hospital query electronically for patients’ health information (e.g., medications, outside encounters) from sources outside of your organization or hospital system?

1. Yes
2. No, but have the capability
3. No, do not have capability

Indicator for electronic data availability:

= 1 (“Yes”) if response 1 was checked for the following survey question

© 2025 Yan AS et al. *JAMA Health Forum*.

= 0 (“No”) otherwise

AHA IT Survey question on electronic availability of data:

When treating a patient that was seen by a provider outside your organization or hospital system, do providers at your hospital routinely have necessary clinical information available electronically (not e-Fax) from outside providers or sources?

1. Yes
2. No

© 2025 Yan AS et al. *JAMA Health Forum*.

eMethods 2. Sample Sizes for Telehealth and HIE Adoption Outcomes by Survey Year

Telehealth Adoption Outcomes

Survey Year	*Sample Size*
2018	2,894
2019	2,803
2020	2,813
2021	2,838
2022	2,793
2023	2,505
Pooled Total	*16,646*

HIE Adoption Outcomes

Survey Year	*Sample Size*
2018	1,992
2019	1,897
2020	1,755
2021	N/A[a]
2022	1,835
2023	1,739
Pooled Total	*9,218*

Notes. *[a]The 2021 AHA Annual IT Survey was not implemented*

© 2025 Yan AS et al. *JAMA Health Forum*.

eTable 1. Marginal Effects of HSA Deprivation on Hospital Telehealth Adoption

	Marginal Effects (MEs) - Logistic Regression Model			
	Telehealth Adoption - Full/Partial vs. None			
	Treatment-Stage		Post-Discharge	
	n = 16,646[a]		n = 16,646[a]	
**Characteristics**	**ME**	**95% CI**	**ME**	**95% CI**
HSA deprivation index national quartile (Ref. = Q1: Least deprived)				
Q2	-0.06***	[-0.09, -0.03]	-0.04*	[-0.07, -0.01]
Q3	-0.07***	[-0.11, -0.04]	-0.04	[-0.08, 0.00]
Q4: Most deprived	-0.06**	[-0.11, -0.02]	-0.06*	[-0.11, -0.01]
Hospital ACO participation (Ref. = Not in an ACO)				
In a Medicare, Medicaid, or private ACO	0.04***	[0.02, 0.07]	0.04**	[0.01, 0.06]
Hospital control type (Ref. = Government)				
Not for profit	0.04*	[0.01, 0.07]	0.01	[-0.02, 0.05]
For profit	-0.08**	[-0.13, -0.03]	-0.15***	[-0.19, -0.10]
Hospital bed size (Ref. = Medium: 50-199 beds)				
Small: <50 beds	-0.07***	[-0.11, -0.04]	-0.04*	[-0.07, -0.01]
Large: >=200 beds	0.07***	[0.04, 0.10]	0.12***	[0.09, 0.16]
Hospital geography (Ref. = Rural)				
Metropolitan	0.01	[-0.03, 0.05]	-0.02	[-0.07, 0.03]
Micropolitan	0.03	[-0.01, 0.07]	0.00	[-0.04, 0.04]
Hospital teaching designation (Ref. = Not a teaching hospital)				
Major teaching hospital	0.16***	[0.11, 0.22]	0.26***	[0.19, 0.33]
Minor teaching hospital	0.02	[-0.01, 0.05]	0.02	[-0.01, 0.05]
% of county population that identifies as a minority race[b]	-0.15***	[-0.22, -0.09]	-0.24***	[-0.32, -0.17]
Survey year fixed effects (Ref. = 2018)				
2019	0.07***	[0.06, 0.08]	0.03***	[0.02, 0.04]
2020	0.20***	[0.18, 0.22]	0.08***	[0.07, 0.10]
2021	0.22***	[0.20, 0.24]	0.10***	[0.08, 0.11]
2022	0.24***	[0.22, 0.26]	0.10***	[0.09, 0.12]
2023	0.24***	[0.22, 0.26]	0.10***	[0.08, 0.11]

***Notes.** The study population consists of non-federal acute care hospitals. MEs were estimated from logistic regressions of hospital telehealth functionality adoption on HSA deprivation, controlling for other hospital characteristics as well as survey year fixed effects. Confidence intervals (CIs) were based on robust standard errors clustered at the hospital level. The HSA deprivation index was derived from the 2021 ADI. Data on telehealth functionality adoption and other hospital characteristics were sourced from the 2018-2023 AHA Annual Survey. Data on county population by race/ethnicity was obtained from the 2023 AHRF. [a]Sample sizes are cumulative over the study period (2018-2023). [b]Minority race is inclusive of all of the following: Hispanic, Non-Hispanic American Indian / Alaskan Native, Non-Hispanic Asian, Non-Hispanic Black, Non-Hispanic Multiracial, Non-Hispanic Native Hawaiian / Pacific Islander, Non-Hispanic Other; and exclusive of Non-Hispanic White. *p<0.05 **p<0.01 ***p<0.001.*

© 2025 Yan AS et al. *JAMA Health Forum*.

eTable 2. Marginal Effects of HRR Deprivation on Hospital Telehealth and HIE Adoption

	Marginal Effects (MEs) - Logistic Regression Model							
	Telehealth Adoption - Full vs. Partial/None				HIE Adoption - Weighted			
	Treatment-Stage		Post-Discharge		Electronic Query Capability		Electronic Availability	
	n = 16,646[a]		n = 16,646[a]		n = 9,218[a]		n = 9,218[a]	
**Characteristics**	**ME**	**95% CI**	**ME**	**95% CI**	**ME**	**95% CI**	**ME**	**95% CI**
HRR deprivation index national quartile (Ref. = Q1: Least deprived)								
Q2	-0.03**	[-0.04, -0.01]	-0.05***	[-0.08, -0.02]	0.02	[-0.00, 0.04]	0.03	[-0.01, 0.06]
Q3	-0.03***	[-0.05, -0.01]	-0.07***	[-0.10, -0.05]	-0.00	[-0.03, 0.02]	0.04	[-0.00, 0.08]
Q4: Most deprived	-0.04***	[-0.06, -0.02]	-0.05**	[-0.08, -0.01]	-0.02	[-0.05, 0.00]	-0.04*	[-0.09, -0.00]
Hospital ACO participation (Ref. = Not in an ACO)								
In a Medicare, Medicaid, or private ACO	0.02**	[0.01, 0.03]	0.03***	[0.02, 0.05]	0.05***	[0.04, 0.07]	0.07***	[0.04, 0.10]
Hospital control type (Ref. = Government)								
Not for profit	0.01	[-0.01, 0.03]	0.01	[-0.01, 0.04]	0.07***	[0.05, 0.09]	0.21***	[0.17, 0.24]
For profit	-0.03*	[-0.05, -0.00]	-0.10***	[-0.13, -0.07]	-0.05*	[-0.09, -0.01]	0.13***	[0.07, 0.20]
Hospital bed size (Ref. = Medium: 50-199 beds)								
Small: <50 beds	-0.02**	[-0.04, -0.01]	-0.04***	[-0.06, -0.02]	-0.04***	[-0.06, -0.02]	0.00	[-0.03, 0.04]
Large: >=200 beds	0.01	[-0.00, 0.03]	0.09***	[0.06, 0.13]	0.04**	[0.01, 0.06]	0.04	[-0.00, 0.07]
Hospital geography (Ref. = Rural)								
Metropolitan	0.02*	[0.01, 0.04]	0.02	[-0.01, 0.06]	0.05***	[0.03, 0.08]	0.14***	[0.10, 0.19]
Micropolitan	0.05***	[0.02, 0.07]	0.05**	[0.01, 0.09]	0.04***	[0.02, 0.07]	0.05	[-0.00, 0.09]
Hospital teaching designation (Ref. = Not a teaching hospital)								
Major teaching hospital	0.09***	[0.05, 0.13]	0.21***	[0.15, 0.27]	0.06***	[0.03, 0.10]	0.13***	[0.08, 0.19]
Minor teaching hospital	0.00	[-0.01, 0.02]	0.01	[-0.01, 0.04]	0.03**	[0.01, 0.05]	0.03*	[0.00, 0.07]
% of county population that identifies as a minority race[b]	-0.04*	[-0.08, -0.00]	-0.15***	[-0.21, -0.09]	-0.09***	[-0.13, -0.04]	-0.17***	[-0.24, -0.09]
Survey year fixed effects (Ref. = 2018)								
2019	0.01***	[0.01, 0.02]	0.02***	[0.01, 0.02]	0.02*	[0.00, 0.04]	-0.02	[-0.04, 0.00]
2020	0.03***	[0.03, 0.04]	0.05***	[0.04, 0.06]	0.07***	[0.05, 0.09]	0.08***	[0.05, 0.10]

© 2025 Yan AS et al. *JAMA Health Forum*.

2021	0.04***	[0.03, 0.04]	0.06***	[0.05, 0.07]	*N/A*[c]	*N/A*[c]	*N/A*[c]	*N/A*[c]
2022	0.04***	[0.03, 0.05]	0.07***	[0.05, 0.08]	0.09***	[0.07, 0.11]	0.12***	[0.10, 0.15]
2023	0.04***	[0.03, 0.05]	0.06***	[0.05, 0.07]	0.07***	[0.05, 0.09]	0.18***	[0.15, 0.20]

Notes. *The study population consists of non-federal acute care hospitals. MEs were estimated from logistic regressions of hospital telehealth and HIE functionality adoption on HRR deprivation, controlling for other hospital characteristics as well as survey year fixed effects. Confidence intervals (CIs) were based on robust standard errors clustered at the hospital level, and non-response weights were applied. The HRR deprivation index was derived from the 2021 ADI. Data on telehealth functionality adoption and other hospital characteristics were sourced from the 2018-2023 AHA Annual Survey. Data on HIE functionality adoption was obtained from the 2018-2020 and 2022-2023 AHA Annual IT Survey. Data on county population by race/ethnicity was obtained from the 2023 AHRF. [a]Sample sizes are cumulative over the study period (2018-2023), and differences are due to varying response rates to the underlying survey questions regarding telehealth services and HIE infrastructure. [b]Minority race is inclusive of all of the following: Hispanic, Non-Hispanic American Indian / Alaskan Native, Non-Hispanic Asian, Non-Hispanic Black, Non-Hispanic Multiracial, Non-Hispanic Native Hawaiian / Pacific Islander, Non-Hispanic Other; and exclusive of Non-Hispanic White. [c]The 2021 AHA Annual IT Survey was not implemented. *p<0.05 **p<0.01 ***p<0.001.*

© 2025 Yan AS et al. *JAMA Health Forum*.

eTable 3. Marginal Effects of HSA Deprivation on Hospital HIE Adoption

	Marginal Effects (MEs) - Logistic Regression Model			
	HIE Adoption - Unweighted			
	Electronic Query Capability		Electronic Availability	
	n = 9,218[a]		n = 9,218[a]	
**Characteristics**	**ME**	**95% CI**	**ME**	**95% CI**
HSA deprivation index national quartile (Ref. = Q1: Least deprived)				
Q2	0.01	[-0.01, 0.03]	0.02	[-0.02, 0.05]
Q3	-0.02*	[-0.05, -0.00]	-0.02	[-0.06, 0.02]
Q4: Most deprived	-0.03**	[-0.06, -0.01]	-0.06*	[-0.11, -0.01]
Hospital ACO participation (Ref. = Not in an ACO)				
In a Medicare, Medicaid, or private ACO	0.05***	[0.04, 0.07]	0.07***	[0.05, 0.10]
Hospital control type (Ref. = Government)				
Not for profit	0.06***	[0.04, 0.08]	0.20***	[0.16, 0.24]
For profit	-0.04*	[-0.08, -0.00]	0.13***	[0.07, 0.19]
Hospital bed size (Ref. = Medium: 50-199 beds)				
Small: <50 beds	-0.03**	[-0.05, -0.01]	0.01	[-0.02, 0.04]
Large: >=200 beds	0.03**	[0.01, 0.05]	0.02	[-0.01, 0.06]
Hospital geography (Ref. = Rural)				
Metropolitan	0.04**	[0.01, 0.06]	0.13***	[0.08, 0.17]
Micropolitan	0.04**	[0.01, 0.06]	0.04	[-0.01, 0.09]
Hospital teaching designation (Ref. = Not a teaching hospital)				
Major teaching hospital	0.06**	[0.02, 0.09]	0.13***	[0.08, 0.19]
Minor teaching hospital	0.02*	[0.00, 0.04]	0.03	[-0.00, 0.06]
% of county population that identifies as a minority race[b]	-0.08***	[-0.12, -0.03]	-0.17***	[-0.25, -0.10]
Survey year fixed effects (Ref. = 2018)				
2019	0.02**	[0.01, 0.04]	-0.02	[-0.04, 0.00]
2020	0.07***	[0.05, 0.09]	0.08***	[0.05, 0.10]
2021	*N/A*[c]	*N/A*[c]	*N/A*[c]	*N/A*[c]
2022	0.09***	[0.07, 0.11]	0.12***	[0.09, 0.15]
2023	0.07***	[0.06, 0.09]	0.18***	[0.15, 0.20]

Notes. *The study population consists of non-federal acute care hospitals. MEs were estimated from logistic regressions of hospital HIE functionality adoption on HSA deprivation, controlling for other hospital characteristics as well as survey year fixed effects. Confidence intervals (CIs) were based on robust standard errors clustered at the hospital level. The HSA deprivation index was derived from the 2021 ADI. Data on HIE functionality adoption was sourced from the 2018-2020 and 2022-2023 AHA Annual IT Survey. Data on other hospital characteristics was obtained from the 2018-2023 AHA Annual Survey. Data on county population by race/ethnicity was obtained from the 2023 AHRF. [a]Sample sizes are cumulative over the study period (2018-2023). [b]Minority race is inclusive of all of the following: Hispanic, Non-Hispanic American Indian / Alaskan Native, Non-Hispanic Asian, Non-Hispanic Black, Non-Hispanic Multiracial, Non-Hispanic Native Hawaiian / Pacific Islander, Non-Hispanic Other; and exclusive of Non-Hispanic White. [c]The 2021 AHA Annual IT Survey was not implemented. *p<0.05 **p<0.01 ***p<0.001.*

© 2025 Yan AS et al. *JAMA Health Forum*.

eTable 4. Hospital and County Characteristics Overall and by Subgroup for 2023

	HSA Deprivation Index National Quartile				
**Characteristics**	**1st Quartile**	**2nd Quartile**	**3rd Quartile**	**4th Quartile**	**Overall**
Hospital treatment-stage telehealth services - *Subgroup totals*	*919****	*944****	*665**	*453*	*2981*
Provides no or some services	834 (90.8%)	876 (92.8%)	633 (95.2%)	442 (97.6%)	2785 (93.4%)
Provides all services	85 (9.2%)	68 (7.2%)	32 (4.8%)	11 (2.4%)	196 (6.6%)
Hospital post-discharge telehealth services - *Subgroup totals*	*919****	*944**	*665*	*453*	*2981*
Provides no or some services	735 (80.0%)	806 (85.4%)	597 (89.8%)	409 (90.3%)	2547 (85.4%)
Provides all services	184 (20.0%)	138 (14.6%)	68 (10.2%)	44 (9.7%)	434 (14.6%)
Hospital electronic data query capability - *Subgroup totals*	*772****	*759****	*529****	*344*	*2404*
Cannot query data from external providers	51 (6.6%)	47 (6.2%)	41 (7.8%)	59 (17.2%)	198 (8.2%)
Can query data from external providers	721 (93.4%)	712 (93.8%)	488 (92.2%)	285 (82.8%)	2206 (91.8%)
Hospital electronic data availability - *Subgroup totals*	*772****	*759****	*529****	*344*	*2404*
No availability of data from external providers	124 (16.1%)	143 (18.8%)	147 (27.8%)	138 (40.1%)	552 (23.0%)
Availability of data from external providers	648 (83.9%)	616 (81.2%)	382 (72.2%)	206 (59.9%)	1852 (77.0%)
Hospital ACO participation - *Subgroup totals*	*806****	*778****	*572***	*353*	*2509*
In a Medicare, Medicaid, or private ACO	593 (73.6%)	527 (67.7%)	363 (63.5%)	186 (52.7%)	1669 (66.5%)
Not in an ACO	213 (26.4%)	251 (32.3%)	209 (36.5%)	167 (47.3%)	840 (33.5%)
Hospital control type - *Subgroup totals*	*919****	*944****	*665****	*453*	*2981*
Not for profit	735 (80.0%)	677 (71.7%)	439 (66.0%)	247 (54.6%)	2098 (70.4%)
For profit	71 (7.7%)	121 (12.8%)	75 (11.3%)	35 (7.7%)	302 (10.1%)
Government	113 (12.3%)	146 (15.5%)	151 (22.7%)	171 (37.7%)	581 (19.5%)
Hospital bed size - *Subgroup totals*	*919****	*944****	*665****	*453*	*2981*
Small: <50 beds	162 (17.6%)	305 (32.3%)	326 (49.0%)	302 (66.6%)	1095 (36.8%)
Medium: 50-199 beds	303 (33.0%)	293 (31.0%)	214 (32.2%)	124 (27.4%)	934 (31.3%)
Large: >=200 beds	454 (49.4%)	346 (36.7%)	125 (18.8%)	27 (6.0%)	952 (31.9%)
Hospital geography - *Subgroup totals*	*919****	*944****	*665****	*453*	*2981*
Metropolitan	812 (88.3%)	687 (72.8%)	275 (41.4%)	65 (14.3%)	1839 (61.7%)
Micropolitan	65 (7.1%)	140 (14.8%)	173 (26.0%)	126 (27.8%)	504 (16.9%)

© 2025 Yan AS et al. *JAMA Health Forum*.

Rural	42 (4.6%)	117 (12.4%)	217 (32.6%)	262 (57.9%)	638 (21.4%)
Hospital teaching designation - *Subgroup totals*	*919****	*944****	*665****	*453*	*2981*
Major teaching hospital	107 (11.6%)	86 (9.1%)	15 (2.3%)	1 (0.2%)	209 (7.0%)
Minor teaching hospital	532 (57.9%)	431 (45.7%)	227 (34.1%)	85 (18.8%)	1275 (42.8%)
Not a teaching hospital	280 (30.5%)	427 (45.2%)	423 (63.6%)	367 (81.0%)	1497 (50.2%)
% County population by race/ethnicity - *Subgroup totals*	*1.00****	*1.00****	*1.00*	*1.00*	*1.00*
Non-Hispanic White	0.56 (0.21)	0.67 (0.20)	0.74 (0.20)	0.74 (0.22)	0.66 (0.22)
Minority race[a]	0.44 (0.21)	0.33 (0.20)	0.26 (0.20)	0.26 (0.22)	0.34 (0.22)

Notes. *The study population consists of non-federal acute care hospitals. The HSA deprivation index was derived from the 2021 ADI. Data on telehealth functionality adoption and other hospital characteristics were sourced from the 2023 AHA Annual Survey. Data on HIE functionality adoption was obtained from the 2023 AHA Annual IT Survey. Data on county population by race/ethnicity was obtained from the 2023 AHRF. Differences in sample sizes are due to varying response rates to the underlying survey questions regarding telehealth services, HIE infrastructure, and ACO participation. [a]Minority race is inclusive of all of the following: Hispanic, Non-Hispanic American Indian / Alaskan Native, Non-Hispanic Asian, Non-Hispanic Black, Non-Hispanic Multiracial, Non-Hispanic Native Hawaiian / Pacific Islander, Non-Hispanic Other; and exclusive of Non-Hispanic White. Student's t-tests were used for significance testing of differences across HSA deprivation index quartiles, with the 4th quartile (most deprived) serving as the reference group: *p<0.05 **p<0.01 ***p<0.001.*

© 2025 Yan AS et al. *JAMA Health Forum*.

eFigure. Maps of Hospital Telehealth and HIE Adoption and HSA Deprivation for 2023

© 2025 Yan AS et al. *JAMA Health Forum*.

eFigure 1A. Map of Treatment-Stage Telehealth Adoption (Y=1/N=0) and HSA Deprivation

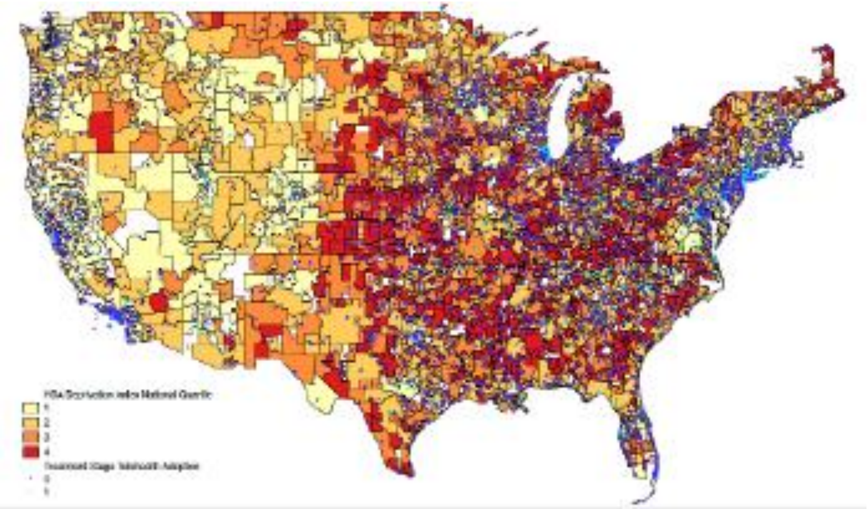

eFigure 1B. Map of Post-Discharge Telehealth Adoption (Y=1/N=0) and HSA Deprivation

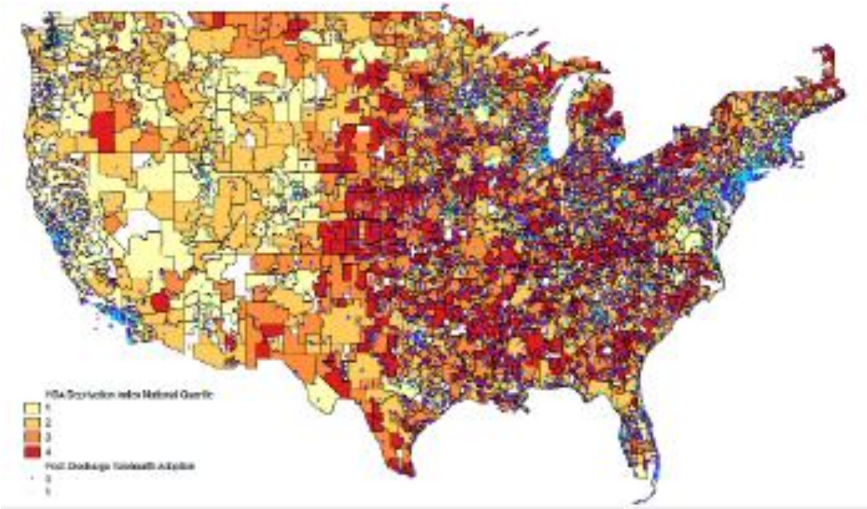

eFigure 1C. Map of Electronic Data Query Capability (Y=1/N=0) and HSA Deprivation

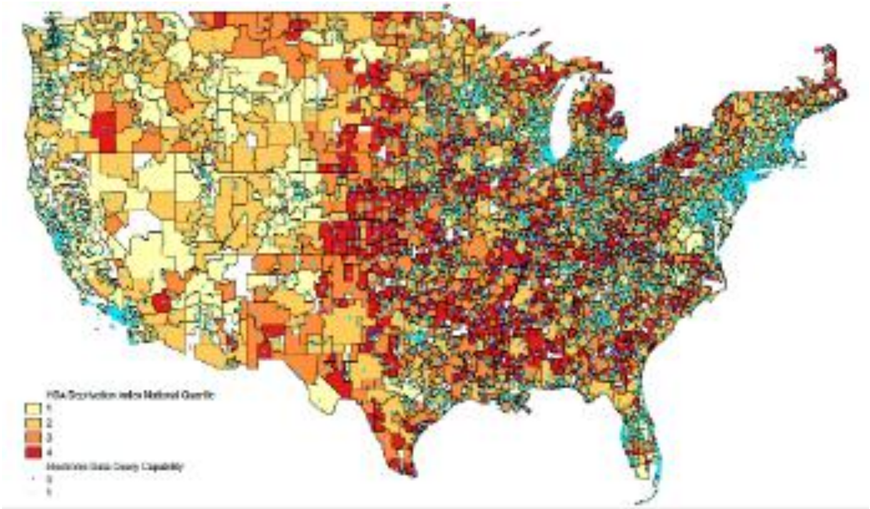

eFigure 1D. Map of Electronic Data Availability (Y=1/N=0) and HSA Deprivation

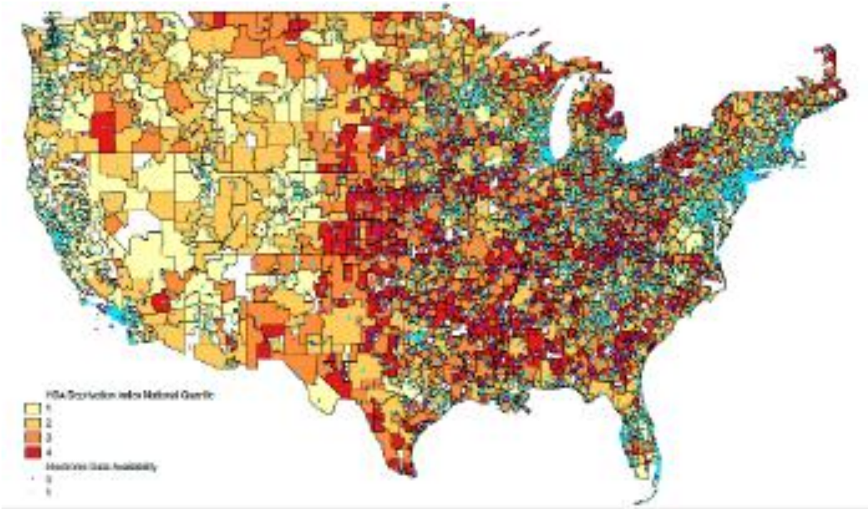

Notes. *The study population consists of non-federal acute care hospitals. The HSA deprivation index was derived from the 2021 ADI. Data on hospital telehealth and HIE functionality adoption were sourced from the 2023 AHA Annual Survey and 2023 AHA Annual IT Survey, respectively.*

© 2025 Yan AS et al. *JAMA Health Forum*.
