# Supplementary material for: Adoption of Health Information Technologies by Area Socioeconomic Deprivation Among US Hospitals
Source: JAMA Health Forum. 2025 Sep 5;6(9):e253035. doi: 10.1001/jamahealthforum.2025.3035 (PMC12413650; doi:10.1001/jamahealthforum.2025.3035)
Supplement: Supplement 2. — Data Sharing Statement [file jamahealthforum-e253035-s002.pdf]

Data Sharing Statement

Yan. Adoption of Health Information Technologies by Area Socioeconomic Deprivation Among US Hospitals. *JAMA Health Forum*. Published September 05, 2025.
doi:10.1001/jamahealthforum.2025.3035

Data
Data available: No

Additional Information
Explanation for why data not available: The American Hospital Association Annual Survey and Information Technology Survey data are restricted by a data use agreement, and cannot be made available. All other data used in our study are publicly available from the following sources: University of Wisconsin Neighborhood Atlas (Area Deprivation Index), Dartmouth Institute Atlas Project for Health Care (hospital service area geographic boundary files), Missouri Census Data Center Geographic Correspondence Engine (ZIP-level population data), and Health Resources and Services Administration Area Health Resources Files.
